# A cross-sectional study of the availability and price of anti-malarial medicines and malaria rapid diagnostic tests in private sector retail drug outlets in rural Western Kenya, 2013

**DOI:** 10.1186/s12936-016-1404-5

**Published:** 2016-07-12

**Authors:** Urbanus Kioko, Christina Riley, Stephanie Dellicour, Vincent Were, Peter Ouma, Julie Gutman, Simon Kariuki, Ahmeddin Omar, Meghna Desai, Ann M. Buff

**Affiliations:** Malaria Control Unit, Ministry of Health, Afya House, Cathedral Road, PO Box 30016, Nairobi, 00100 Kenya; Field Epidemiology and Laboratory Training Programme, Ministry of Health, Afya House, Cathedral Road, PO Box 30016, Nairobi, 00100 Kenya; Emory Rollins School of Public Health, 1518 Clifton Road NE, Atlanta, GA 30322 USA; Liverpool School of Tropical Medicine, Pembroke Place, Liverpool, Merseyside L3 5QA UK; KEMRI, Centre for Global Health Research, Box 1578, Kisumu, 40100 Kenya; Malaria Branch, Division of Parasitic Diseases and Malaria, Center for Global Health, CDC, 1600 Clifton Rd NE, Mailstop A-06, Atlanta, GA 30333 USA; U.S. President’s Malaria Initiative, United Nations Avenue, Village Market, PO Box 606, Nairobi, 00621 Kenya

**Keywords:** Malaria, Anti-malarials, Diagnostic tests, Private sector, Kenya

## Abstract

**Background:**

Although anti-malarial medicines are free in Kenyan public health facilities, patients often seek treatment from private sector retail drug outlets. In mid-2010, the Affordable Medicines Facility-malaria (AMFm) was introduced to make quality-assured artemisinin-based combination therapy (ACT) accessible and affordable in private and public sectors.

**Methods:**

Private sector retail drug outlets stocking anti-malarial medications within a surveillance area of approximately 220,000 people in a malaria perennial high-transmission area in rural western Kenya were identified via a census in September 2013. A cross-sectional study was conducted in September–October 2013 to determine availability and price of anti-malarial medicines and malaria rapid diagnostic tests (RDTs) in drug outlets. A standardized questionnaire was administered to collect drug outlet and personnel characteristics and availability and price of anti-malarials and RDTs.

**Results:**

Of 181 drug outlets identified, 179 (99 %) participated in the survey. Thirteen percent were registered pharmacies, 25 % informal drug shops, 46 % general shops, 13 % homesteads and 2 % other. One hundred sixty-five (92 %) had at least one ACT type: 162 (91 %) had recommended first-line artemether-lumefantrine (AL), 22 (12 %) had recommended second-line dihydroartemisinin-piperaquine (DHA-PPQ), 85 (48 %) had sulfadoxine-pyrimethamine (SP), 60 (34 %) had any quinine (QN) formulation, and 14 (8 %) had amodiaquine (AQ) monotherapy. The mean price (range) of an adult treatment course for AL was $1.01 ($0.35–4.71); DHA-PPQ was $4.39 ($0.71–7.06); QN tablets were $2.24 ($0.12–4.71); SP was $0.62 ($0.24–2.35); AQ monotherapy was $0.42 ($0.24–1.06). The mean AL price with or without the AMFm logo did not differ significantly ($1.01 and 1.07, respectively; p = 0.45). Only 17 (10 %) drug outlets had RDTs; 149 (84 %) never stocked RDTs. The mean RDT price was $0.92 ($0.24–2.35).

**Conclusions:**

Most outlets never stocked RDTs; therefore, testing prior to treatment was unlikely for customers seeking treatment in the private retail sector. The recommended first-line treatment, AL, was widely available. Although SP and AQ monotherapy are not recommended for treatment, both were less expensive than AL, which might have caused preferential use by customers. Interventions that create community demand for malaria diagnostic testing prior to treatment and that increase RDT availability should be encouraged.

## Background

In Kenya, malaria accounts for more than 20 % percent of outpatient visits, 19 % of hospital admissions, 3–5 % of hospital deaths and is a leading cause of mortality in children less than 5 years of age [1, 2]. The World Health Organization (WHO) and Kenya national malaria treatment guidelines recommend prompt diagnosis with a parasitological test (i.e., microscopy or malaria rapid diagnostic test [RDT]) and treatment with an effective anti-malarial medicine as the cornerstone of malaria case management [3, 4]. Since 2004, the recommended first-line artemisinin-based combination therapy (ACT) for uncomplicated malaria in Kenya has been artemether-lumefantrine (AL); AL is provided free in public health facilities. However, studies have shown that between 17–83 % of persons with fever are first treated with medicine purchased from private sector retail drug outlets rather than the formal health sector [5, 6]. People seeking treatment from private retail drug outlets are less likely to receive anti-malarial medicines recommended in the national malaria treatment guidelines [4–6]. In addition, private retail drug outlets historically have been less likely to offer diagnostic testing for malaria prior to selling anti-malarial medicines to customers, which is contrary to national malaria treatment guidelines [4, 7].

An important determinant of prompt and effective treatment is the availability of the recommended anti-malarial medicines in private retail drug outlets. The Affordable Medicine Facility—malaria (AMFm) programme was introduced on a pilot basis in eight countries in 2010, including Kenya [7]. The AMFm provided quality-assured ACT (QAACT) to wholesalers at a heavily-subsidized cost with the objectives of increasing ACT availability, affordability, use and decreasing availability (i.e., “crowding-out”) of treatments no longer recommended such as sulfadoxine-pyrimethamine (SP), chloroquine and oral artemisinin monotherapies [7, 8]. The packaging of AMFm-subsidized QAACT is marked with a distinctive green leaf logo for easy identification. In Kenya, the AMFm subsidy targeted primarily quality-assured AL in both the public and private sectors. From September 2010 to December 2011, the AMFm pilot largely met the objectives of increasing availability and achieving an initial target price of $0.50 for QAACTs to the consumer; the availability increased from 21 to 60 % in retail drug outlets, and the price decreased from a median of $2.63 to $0.58 overall and to $0.46 in rural areas [7]. In Kenya, the original AMFm subsidy ended in 2012. In 2013, AMFm was extended under a revised subsidy scheme, which decreased the subsidy to wholesalers and increased the target price of QAACTs to $1.00 to the consumer. The target price of $1.00 was across QAACT packages for both children and adults.

In 2013, a census was conducted of all private sector retail drug outlets in a surveillance area covering approximately 220,000 people in Siaya County, western Kenya, as part of a larger study to assess prescribing behaviours and knowledge of malaria in pregnancy treatment guidelines [9]. Following the census, a cross-sectional survey was conducted to determine the availability and price of anti-malarial medicines and RDTs in private retail drug outlets. Characteristics of the surveyed retail drug outlets and personnel and the availability and price of anti-malarial medicines and RDTs are described.

## Methods

### Study area

A cross-sectional study was conducted from September to October 2013 in the Kenya Medical Research Institute and Centers for Disease Control and Prevention’s Health and Demographic Surveillance System (KEMRI/CDC HDSS) in Siaya County, western Kenya. The HDSS covers approximately 700 square kilometres and includes parts of three sub-counties, Gem, Siaya and Rarieda, with an estimated total population of 220,000 or approximately one-fifth of the county population [10]. In 2012, an estimated 38 % of the population in Siaya County was below the poverty level and lived on less than $1.25 per day [11]. Malaria transmission is perennially high with peaks in May–July and October–November coinciding with the end of seasonal rains. Overall parasitaemia prevalence in the HDSS population was 34.5 % in 2013 (unpublished data, Simon Kariuki, KEMRI and Meghna Desai, CDC). The HDSS hosts numerous studies on malaria and other diseases and has been described in detail elsewhere [10].

### Data collection

A census was undertaken to identify all private sector, retail drug outlets in the HDSS boundaries; local HDSS staff worked with community members and leaders to ensure all possible establishments were initially considered for study inclusion. A private retail drug outlet was any registered pharmacy, informal drug shop, general shop, homestead or other establishment that sold anti-malarial medications or malaria RDTs. The type of retail drug outlet was categorized by observation and self-report. Registered pharmacy status was ascertained by self-report from the person on duty at the establishment. General shops sold medications and other non-health related goods. The category of “other” drug outlets included mobile vendors, veterinary shops stocking human and animal medicines and shops operated by for-profit community-based organisations. The daily hours of operations and patient volume data were reported by the person on duty at the establishment. A structured, standardized questionnaire was administered to proprietors of the retail drug outlets who agreed to participate in the survey; data collected included retail drug outlet characteristics, availability, type, and pricing of anti-malarial medicines and RDTs in stock on the day of the survey and within the last 3 months.

Anti-malarial medications and malaria RDTs were considered “in stock” if the retail drug outlet had at least one unit available for sale to customers on the day of the survey. Anti-malarial medicines and malaria RDTs were considered “ever stocked” if the retail drug outlet had sold malaria commodities within the last three months. All anti-malarial medicines and RDTs reported during the survey were visually verified by study staff; artemisinin-based combinations were systematically examined for the AMFm logo. All anti-malarial medicine prices were based on an adult-equivalent treatment dose unless otherwise indicated. An adult-equivalent treatment dose was defined as the number of milligrams (mg) of an anti-malarial medicine needed to treat a 60-kg adult. The local price in Kenya shillings was converted to U.S. dollars using the October 2013 exchange rate of 85 Kenya shillings to $1.00. All retail drug outlet data were collected via electronic personal data assistants in the field.

### Data analysis

Data analysis was restricted to drug outlets that stocked any anti-malarial medicine or malaria RDT on the day of the survey or within the last 3 months. Data were entered, cleaned and analysed using Epi-Info Version 7 (CDC, Atlanta, GA, USA), Microsoft Access and Excel 2010 (Microsoft, Seattle, WA, USA) and SAS version 9.2 (SAS Institute Inc., Cary, NC). Univariate analysis was performed to determine the frequencies and proportions of the characteristics of retail drug outlets, employees and malaria commodities.

### Ethics, consent and permissions

A letter stating the purpose of the study was obtained from the Siaya County Director of Health. Local HDSS staff explained the purpose of the study to drug outlet personnel and provided a copy of the letter; verbal consent was obtained in the local language prior to administration of the questionnaire. No personal identifying information was collected from study participants. The study was approved by the institutional review boards of Kenyatta National Hospital/University of Nairobi (#P468/09/2013), Nairobi, Kenya), KEMRI (#2563, Nairobi, Kenya) and Liverpool School of Tropical Medicine (#13.18, Liverpool, UK). The study underwent human subjects review at CDC and was approved as non-engagement in human subject research.

## Results

### Retail drug outlet characteristics

The census identified a total of 181 private retail drug outlets that stocked anti-malarial medicines within the last 3 months in the KEMRI/CDC HDSS; 179 agreed to participate in the survey and were included in the analysis. Among the retail drug outlets, 47 % (n = 83) were general shops, 25 % (n = 45) informal drug shops, 13 % (n = 24) registered pharmacies, 13 % (n = 23) homesteads and 2 % (n = 4) other (Table 1). Drug outlets were open for a mean of 12.6 h per day with a range from 6 h in general shops to over 17 h in homesteads. Drug outlets served an estimated mean of 25.7 and a median of 10.0 customers per day (range 1–250). Registered pharmacies served the greatest number of customers with an estimated mean of 74 and a median of 50 customers per day (range 10–250) compared to homesteads serving an estimated mean 5.6 and a median of five customers per day (range 1–10).

**Table 1:** Characteristics of private sector retail drug outlets in Siaya County, Kenya—2013

	Drug-outlet type		Personnel employed			Business open (hours per day)		Estimated daily customers^a^		
	n	(%)	n	Mean	Range	Mean	Range	n	Mean	Range
Registered pharmacy	24	(13.4)	47	2.0	1–4	12.1	9.5–14.5	21	74.0	10–250
Informal drug shop	45	(25.1)	62	1.4	1–2	11.2	7.0–16.0	44	34.4	3–200
General shop	83	(46.4)	123	1.5	1–2	13.1	6.0–16.0	83	15.0	2–100
Homestead	23	(12.8)	26	1.1	1–2	14.2	8.0–17.0	23	5.6	1–10
Other^c^	4	(2.2)	5	1.3	1–2	11.6	8.0–15.0	4	12.5	10–20
Total	179	(100.0)^b^	263	1.5	1–4	12.6	6.0–17.0	175	25.7	1–250

^a^N = 175 drug outlets; four removed from analysis due to estimates >3 standard deviations above mean (outliers)

^b^Total equals slightly less than 100 % due to rounding

^c^Other category included mobile vendors, veterinary shops stocking human and animal medicines and shops operated by for-profit community-based organisations

### Retail drug outlet personnel characteristics

There were a total of 263 employees with an average of 1.5 (range 1–4) employees per retail drug outlet (Table 1). Registered pharmacies had the greatest mean number of employees (2.0; range: 1–4), and homesteads had the fewest (1.1; range 1–2). Overall, 28 % of drug-outlet personnel reported completing primary school, 37 % secondary school and 33 % had at least some higher education. Registered pharmacies had the greatest proportion at 40 % (n = 47) of employees with at least some higher pharmacy-specific education.

### Availability of anti-malarial medicines and RDTs

Table 2 shows the availability of anti-malarial medicines and RDTs across retail drug-outlet types. At least one ACT, SP and quinine formulations were stocked across all retail drug-outlet types. Overall, 92 % (165/179) of retail drug outlets stocked at least one ACT. Of the 165 ACT-stocking outlets 98 % (n = 162) stocked AL; only 13 % (n = 22) stocked dihydroartemisinin-piperaquine (DHA-PPQ), and 10 % (n = 16) stocked other forms of ACT (i.e., artesunate-amodiaquine or artemisinin-piperaquine). Among the 201 AL packages at 162 drug outlets observed by study staff, 66 % (n = 132) had the AMFm logo. Packages of AL with the AMFm logo were found across all retail drug-outlet types. Quinine was stocked in 34 % (n = 60) of drug outlets; informal drug shops (78 %) and registered pharmacies (71 %) were most likely to stock quinine. Among the retail drug outlets stocking quinine, 62 % stocked the parenteral formulation, 48 % stocked tablets and 32 % stocked suspension. SP was stocked in 48 % (n = 85) of retail drug outlets; registered pharmacies (75 %) and informal drug shops (76 %) were most likely to have SP. Only 8 % (n = 14) of retail drug outlets stocked amodiaquine monotherapy; no registered pharmacy stocked it. Artemether parenteral formulation for the treatment of severe malaria was stocked by 5 % (n = 9) of retail drug outlets, and 25 % (n = 6) of registered pharmacies but no general shops or homesteads stocked parenteral artemether. No retail drug outlets stocked chloroquine or other artemisinin-based monotherapy formulations, such as oral or parenteral artesunate. Only 10 % (n = 17) of retail drug outlets had RDTs in stock at the time of the study; one-third (n = 8) of registered pharmacies stocked RDTs. The majority (84 %, n = 149) of retail drug outlets had never stocked RDTs.

**Table 2:** Availability of anti-malarial medicines and malaria rapid diagnostic tests by drug-outlet type in Siaya County, Kenya—2013

	Total drug outlets n = 179 column (%)		Registered pharmacies n = 24 column (%)		Informal drug shops n = 45 column (%)		General shops n = 83 column (%)		Homesteads n = 23 column (%)		Other n = 4 column (%)	
Any ACT	165	(92.2)	23	(95.8)	44	(97.8)	73	(88.0)	21	(91.3)	4	(100.0)
Artemether-lumefantrine	162	(90.5)	22	(91.7)	43	(95.6)	72	(86.7)	21	(91.3)	4	(100.0)
Dihydroartemisinin-piperaquine	22	(12.3)	15	(62.5)	6	(13.3)	1	(1.2)	–		–	
Artesunate-amodiaquine	12	(6.7)	3	(12.5)	5	(11.1)	3	(3.6)	1	(4.3)		
Artemisinin-piperaquine	4	(2.2)	3	(12.5)	1	(2.2)	–		–		–	
Any quinine formulation	60	(33.5)	17	(70.8)	35	(77.8)	2	(2.4)	3	(13.0)	3	(75.0)
Tablets	29	(48.3)	9	(37.5)	15	(33.3)	1	(1.2)	2	(8.7)	2	(50.0)
Parenteral	37	(61.7)	12	(50.0)	19	(42.2)	2	(2.4)	1	(4.3)	3	(75.0)
Suspension	19	(31.7)	4	(16.7)	15	(33.3)	–		–		–	
Sulfadoxine-pyrimethamine	85	(47.5)	18	(75.0)	34	(75.6)	20	(24.1)	10	(43.5)	3	(75.0)
Amodiaquine	14	(7.8)	–		3	(6.7)	10	(12.0)	1	(4.3)	–	
Artemether, parenteral	9	(5.0)	6	(25.0)	2	(4.4)	–		–		1	(25.0)
Artemether-lumefantrine	n = 201^a^		n = 28		n = 56		n = 84		n = 29		n = 4	
AMFm green leaf logo	132	(65.7)	20	(71.4)	46	(82.1)	50	(59.5)	13	(44.8)	4	(100.0)
Artesunate-amodiaquine	n = 12		n = 3		n = 5		n = 3		n = 1			
AMFm green leaf logo	11	(91.7)	2	(66.7)	5	(100)	3	(100)	1	(100)	–	
Malaria rapid diagnostic tests	n = 177^b^		n = 24		n = 45		n = 82		n = 22		n = 4	
Available on survey day	17	(9.6)	8	(33.3)	5	(11.1)	1	(1.2)	3	(13.6)	–	
Never stocked	149	(84.2)	14	(58.3)	34	(75.6)	81	(98.8)	19	(86.4)	1	(25.0)

*ACT* artemisinin-based combination therapy; *AMFm* Affordable Medicines Facility-malaria program

^a^Multiple brands of anti-malarial medications were stocked at some drug outlets

^b^Data missing for two drug outlets; drug outlets excluded from analysis

### Price of anti-malarial medicines and RDTs

Table 3 shows detailed prices stratified by retail drug-outlet type. The price of anti-malarial medicines differed across retail drug-outlet types and brands of medicine; prices were calculated on an adult-equivalent treatment dose for uncomplicated malaria except for parenteral formulations, which were per vial. The AMFm logo was only identified on two types of ACTs, AL and artesunate-amodiaquine. The overall mean price of AL, the recommended first-line treatment, was $1.01 and median price was $0.94 (range $0.35–4.71). There was no significant difference in the mean price of AL with the AMFm logo (n = 132) at $1.01 (range $0.35–3.53) compared to without the logo (n = 69) at $1.07 (range $0.47–4.71; p = 0.45). Registered pharmacies had the highest mean price at $1.28 and largest price range for AL. The recommended second-line ACT, DHA-PPQ, was substantially more expensive than AL with an overall mean price of $4.39 and median price of $4.14 (range $0.71–7.06). Artesunate-amodiaquine was the least expensive ACT with an overall mean and median price of $0.71 (range $0.59–1.18); all packages of artesunate-amodiaquine except one had the AMFm logo. Artemisinin-piperaquine was the most expensive ACT with an average price of $5.53 and median price of $5.47 (range $5.29–5.88).

**Table 3:** Price in U.S. dollars of anti-malarial medicines and malaria rapid diagnostic tests by drug-outlet type in Siaya County, Kenya—2013

	Overall median	Overall mean (range)	Registered pharmacy mean (range)	Informal shop mean (range)	General shop mean (range)	Homestead mean (range)	Other mean (range)
	Price in U.S. dollars ($)^a^						
Artemisinin-based combination therapy							
Artemether-lumefantrine	0.94	1.01 (0.35–4.71)	1.28 (0.35–4.71)	1.00 (0.35–2.94)	0.92 (0.35–1.76)	1.09 (0.71–1.76)	1.06 (0.94–1.41)
Dihydroartemisinin-piperaquine	4.14	4.39 (0.71–7.06)	4.38 (0.71–7.06)	4.45 (3.53–5.29)	4.12 (3.53–4.71)	–	–
Artesunate-amodiaquine	0.71	0.71 (0.59–1.18)	0.63 (0.59–0.71)	0.73 (0.59–1.18)	0.71 (NA^b^)	0.82 (NA)	–
Artemisinin-piperaquine	5.47	5.53 (5.29–5.88)	5.61 (5.29–5.88)	5.29 (NA)	–	–	–
Quinine formulations							
Tablets	2.31	2.24 (0.12–4.71)	2.93 (1.09–4.71)	1.86 (0.12–3.53)	2.82 (NA)	1.62 (0.59–2.65)	2.35 (0.88–3.81)
Parenteral (600 mg/2 ml vial)	0.35	0.48 (0.24–1.06)	0.50 (0.24–1.06)	0.51 (0.29–1.06)	0.41 (0.35–0.47)	0.41 (NA)	0.33 (0.29–0.35)
Paediatric suspension^c^ (50 mg/5 ml)	0.82	0.94 (0.53–2.35)	0.75 (0.53–0.94)	1.00 (0.53–2.35)	0.82 (NA)	–	–
Sulfadoxine-pyrimethamine	0.47	0.62 (0.24–2.35)	0.76 (0.29–1.76)	0.68 (0.35–2.35)	0.39 (0.24–0.82)	0.50 (0.35–0.82)	0.91 (0.35–1.76)
Amodiaquine monotherapy	0.35	0.42 (0.24–1.06)	–	0.33 (0.29–0.35)	0.45 (0.24–1.06)	0.35 (NA)	–
Artemether, parenteral (80 mg/ml vial)	1.18	1.53 (0.47–4.12)	1.75 (0.94–4.12)	0.82 (0.47–1.18)	–	–	1.18 (NA)
Malaria rapid diagnostic tests	0.59	0.92 (0.24–2.35)	1.02 (0.35–2.35)	0.87 (0.24–2.35)	–	0.71 (0.35–1.18)	–

Price per standard adult-equivalent treatment dose for all medicines except parenteral and paediatric suspension formulations

^a^All prices in U.S. dollars (USD) converted from Kenya Shillings (KES) based on exchange rate in October 2013 (1 USD = 85 KES)

^b^ *NA* not applicable. Only one price for the medicine formulation; no range reported

^c^Price analysis for most common formulation only, which represented 87.5 % (21/24) of total

The mean price of quinine varied across retail drug-outlet types and with formulation. The overall mean price of quinine tablets was $2.24 (range $0.12–4.71). The parenteral quinine formulation per 600 mg/2 ml vial had a mean price of $0.48 (range $0.24–1.06). The most common quinine paediatric suspension formulation (50 mg/5 ml) had an overall mean price of $0.94 (range $0.53–2.35). The overall mean price of SP was $0.62 (range $0.24–2.35); SP was most expensive in other outlets and least expensive in general shops. The overall mean price of amodiaquine monotherapy was $0.42 (range $0.24–1.06) and artemether parenteral formulation (80 mg/ml vial) was $1.53 (range $0.47–4.12).

The overall mean price of malaria RDTs was $0.92 and median price was $0.59 (range $0.24–2.35). Malaria RDTs were most expensive in registered pharmacies and least expensive in homesteads. The total mean price of following national malaria treatment guidelines at a retail drug outlet (i.e., diagnosis of uncomplicated malaria with an RDT and treatment with AL with the AMFm logo) was $1.93 (range $0.59–7.06).

## Discussion

The study demonstrates widespread availability of effective anti-malarial medicines, including the recommended first-line AL, across retail drug outlets in rural Siaya County, western Kenya. The mean price of quality-assured AL with the AMFm logo was consistent with the 2013 revised target price of $1.00, and quality-assured artesunate-amodiaquine with the AMFm logo was priced 29 % less than the target price to the customer. The data demonstrate that the 2013 extension of the AMFm subsidy under the revised scheme has continued the trend of increasing availability and affordability of ACT in the retail sector as reported by the AMFm Independent Evaluation Team in 2012 [7]. The recommended first-line ACT, AL, was available in over 90 % of drug outlets surveyed. In less than 2 years from December 2011 to October 2013, ACT availability increased from 60 % to over 90 % in retail drug outlets in rural western Kenya [7]. Although not part of the AMFm program in Kenya, DHA-PPQ is the recommended anti-malarial medicine for malaria treatment failures [4]. Treatment failures with AL are relatively uncommon in western Kenya, but re-infection is common [12, 13]. Only a minority of drug outlets stock DHA-PPQ and the price is over four times greater than AL; therefore, customers are probably less likely to purchase DHA-PPQ as a first-line treatment.

Although no retail drug outlets stocked chloroquine, almost half of all drug outlets continue to sell SP for treatment of uncomplicated malaria and a small percentage of drug outlets sell amodiaquine monotherapy. On average, SP is one-third less expensive than AL, and amodiaquine monotherapy is almost two-thirds less. Because SP and amodiaquine monotherapy are substantially less expensive than AL, customers might preferentially choose one of these non-recommended medications. Evidence from neighboring Busia County in western Kenya demonstrates that when adults are uncertain that malaria is the true cause of their illness, they tend to choose the lowest-priced anti-malarial medicine first from retail drug outlets [6]. Use of SP for malaria case management has not been recommended in Kenya since 2004 due to widespread resistance [14–16]. Therefore, customers who purchase SP are likely to be using an ineffective treatment, which can lead to delays in obtaining the correct treatment and increasing the potential for progression to severe malaria, particularly in children and pregnant women.

In Kenya, SP is recommended only for intermittent preventive treatment of malaria in pregnancy (IPTp) in malaria-endemic areas, including Siaya County and is generally delivered as part of the antenatal care (ANC) package [4]. Retail drug outlets might stock SP for sale to private health facilities that provide ANC services or for women to purchase in the event of SP stock outs at public ANC clinics. However, it appears likely that SP is being sold to customers for treatment of uncomplicated malaria. Riley et al. demonstrated that retail drug-outlet personnel sold SP as treatment to 11 % of simulated clients who asked for treatment due to signs and symptoms consistent with malaria, and almost half of retail drug-outlet providers incorrectly reported that SP could be used for treatment [9]. In addition, more drug outlets sell SP (48 %) than quinine (34 %), which is the first-line treatment for malaria in pregnancy during the first trimester.

Malaria RDTs are not widely available in retail drug outlets in western Kenya. Under current Kenya regulations, only registered pharmacies are licensed to sell medications and point-of-service diagnostic testing is not permitted. A pilot project to allow pharmacists to conduct diagnostic testing with malaria RDTs in registered pharmacies, under a Government of Kenya waiver, was conducted in three coastal counties in 2014 [17]. Evidence from the pilot suggested that after a package of malaria RDT interventions was introduced, the quality of malaria RDT services was comparable between private health facility providers and registered pharmacists; in addition, access and informed demand for malaria testing before treatment led to testing of 90 % of clients in both private health facilities and registered pharmacies [17]. Data from this and over a dozen other private sector malaria RDT pilot projects is informing the discussion of changing national policies and regulations to allow point-of-service diagnostic testing at registered pharmacies in Kenya and other malaria-endemic countries to improve malaria case management practices [17–19].

However, in this rural western Kenya study area, only 13 % of private drug outlets were registered pharmacies. Policy and regulation changes would only affect registered pharmacies, which comprise a minority of drug outlets where rural communities seek care. As a result, customers are unlikely to be tested for malaria prior to buying an anti-malarial medicine in drug outlets, which is contrary to national treatment guidelines and increases the likelihood of over-prescribing [4]. National treatment guidelines recommend parasitological testing prior to treatment to improve the rational use of relatively expensive ACT, prevent resistance to artemisinin, and ensure patients are promptly and correctly treated for fever-related illness [4]. Based on significant case management improvements when malaria RDTs were introduced into registered drug shops in Uganda, Mbonye et al. developed policy recommendations to encourage active and expanded registration of drug outlets so more drug outlets could benefit from potential changes in policy and regulation that would allow point-of-service diagnostic testing for malaria [18, 20]. Evidence from western Kenya suggests that drug retailers are interested in stocking malaria RDTs because they see the potential for increased business and an opportunity to increase both retailer and customer confidence in the diagnosis and treatment of malaria [21]. Currently, only 10 % of retail drug outlets surveyed could offer both malaria RDTs and ACT to customers.

In addition, interventions that encourage drug outlets to stock and appropriately use malaria RDTs, such as training and supervision packages combined with commodity subsidies and provider incentives, are likely to increase point-of-service diagnostic testing [6, 17, 19, 22]. In a simulated-client survey conducted in a sample of the drug outlets stocking RDTs from this study, only 17 % of clients were offered an RDT (unpublished data, Christina Riley, Emory Rollins School of Public Health). However, testing before treatment for malaria is common in the public health sector throughout Kenya. The nationally-representative malaria quality-of-care survey conducted in September 2014 showed that 91 % of public (i.e., Ministry of Health) and non-profit (i.e., non-governmental organisation or faith-based) facilities had either malaria RDTs or functional microscopy services available, and 76 % had AL in stock [23]. Among febrile patients, two-thirds were tested for malaria; of those with a positive malaria test result, 88 % received the recommended treatment and only 9 % with a negative malaria test received anti-malarial medicines at surveyed health facilities [23]. Therefore, the majority of patients with malaria who access care in the public health sector receive care in line with the national malaria guidelines. Strategies that address community trust in the public health sector and encourage preferential use of public and non-profit health facilities should be considered to improve the overall case management of febrile illness and malaria.

## Conclusions

In Siaya County, western Kenya, the majority of retail drug outlets did not stock RDTs in 2013; therefore, testing prior to treatment in accordance with the national treatment guidelines was unlikely for customers seeking anti-malarial medicines first in the retail sector. In 2013, the recommended first-line treatment, AL, was widely available across drug-outlet types in rural western Kenya. Although SP and amodiaquine monotherapy are not recommended for treatment, both were substantially less expensive than AL, which might have caused preferential use by customers. Interventions that increase the availability and affordability of malaria RDTs in the retail sector and create customer demand for testing prior to treatment should be encouraged to improve community malaria case management practices.

## Abbreviations

AMFm: Affordable Medicines Facility-malaria
AQ: amodiaquine
ANC: antenatal care
AL: artemether-lumefantrine
ACT: artemisinin-based combination therapy
CDC: Centers for Disease Control and Prevention
DHA-PPQ: dihydroartemisinin-piperaquine
HDSS: health and demographic surveillance system
IPTp: intermittent preventive treatment of malaria in pregnancy
KEMRI: Kenya Medical Research Institute
QAACT: quality-assured artemisinin-based combination therapy
QN: quinine
RDT: rapid diagnostic test
SP: sulfadoxine-pyrimethamine
WHO: World Health Organization
